# Cortical encoding of pitch: Recent results and open questions

**DOI:** 10.1016/j.heares.2010.04.015

**Published:** 2011-01

**Authors:** Kerry M.M. Walker, Jennifer K. Bizley, Andrew J. King, Jan W.H. Schnupp

**Affiliations:** Dept. of Physiology, Anatomy and Genetics, Sherrington Building, University of Oxford, Parks Road, Oxford OX1 3PT, United Kingdom

**Keywords:** A1, primary auditory cortex, F0, fundamental frequency, fMRI, functional magnetic resonance imaging, HG, Heschl’s gyrus, IRN, iterated rippled noise, MEG, magnetoencephalography, SAM, sinusoidally amplitude modulated

## Abstract

It is widely appreciated that the key predictor of the pitch of a sound is its periodicity. Neural structures which support pitch perception must therefore be able to reflect the repetition rate of a sound, but this alone is not sufficient. Since pitch is a psychoacoustic property, a putative cortical code for pitch must also be able to account for the relationship between the amount to which a sound is periodic (i.e. its temporal regularity) and the perceived pitch salience, as well as limits in our ability to detect pitch changes or to discriminate rising from falling pitch. Pitch codes must also be robust in the presence of nuisance variables such as loudness or timbre. Here, we review a large body of work on the cortical basis of pitch perception, which illustrates that the distribution of cortical processes that give rise to pitch perception is likely to depend on both the acoustical features and functional relevance of a sound. While previous studies have greatly advanced our understanding, we highlight several open questions regarding the neural basis of pitch perception. These questions can begin to be addressed through a cooperation of investigative efforts across species and experimental techniques, and, critically, by examining the responses of single neurons in behaving animals.

## 1 Introduction

Many objects in nature, including vocal cords, can enter into regular vibrations and create pressure waves in the air that repeat periodically over a certain time interval. Our brains interpret these periodic pressure waves as a sound with pitch. The American National Standards Institute (1994) defined pitch as “that auditory attribute of sound according to which sounds can be ordered on a scale from low-to-high”. This definition emphasizes only one dimension of pitch perception – pitch *height*. We can also classify the pitch *chroma*, which is constant across pitch height differences of an octave. For example, the “middle C” (C_4_) and higher C’s (C_5_, C_6_, etc) on a Western musical scale differ in pitch height but all collectively describe a single pitch class and therefore share the same pitch chroma. Additionally, the perceptual strength, or *salience*, of pitch can differ across sounds that have the same pitch height and chroma. The current paper focuses on how listeners perceive pitch along the height and salience dimensions, and how this information is encoded by neurons in auditory cortex.

In order for listeners to use periodicity cues effectively, the pitch associated with a given periodicity of sound should be independent of other acoustical features, such as loudness or timbre. To a first approximation, pitch perception exhibits such invariance. This allows us to recognize a melody whether it is played on a violin or a piano, or sung by a human or songbird, or even if it is generated artificially by a computer. Despite much recent progress, there remains a great deal of uncertainty regarding how neurons in auditory cortex encode pitch, but such mechanisms should be able to account for a number of perceptual phenomena, including: a) the correlation of pitch salience with relevant acoustical features such as temporal regularity and harmonic spacing; b) listeners’ ability to detect differences in the pitch of sounds with different repetition rates; c) listeners’ ability to order the pitch of sounds along a low-to-high scale; and d) the generalization of pitch perception across sounds that differ in other, irrelevant perceptual attributes. This is not an exhaustive list of criteria for identifying the neural substrate for pitch perception, but meeting them would advance our current understanding in this field.

Here, we explore evidence regarding how the activity of auditory cortical neurons contributes to pitch judgments in humans and animals. We begin by discussing the acoustical properties that determine pitch, and we then address the validity of animal models of pitch perception. Next, we will briefly review the representation of pitch cues in auditory subcortical structures. Finally, we describe studies of the relationships between auditory cortical activity and listeners’ ability to discriminate, order, and generalize the pitch of sounds.

## 2 Acoustical correlates of pitch

Pitch is related to the periodicity of sounds – the manner in which their waveform repeats throughout time. Sounds with a faster repetition rate evoke a “higher” pitch, and sounds that are more temporally regular evoke a more salient pitch, but this phenomenon only holds for repetition rates between approximately 30–5000 Hz (Krumbholz et al., 2000; Semal and Demany, 1990). Human listeners can hear frequencies of sound above the upper pitch limit, but they have difficulties recognizing melodies beyond this range. As an alternative to conceptualizing periodicity as a temporal property of sound waves, we can also describe pitch-evoking sounds in terms of their harmonic structure. When viewed in the spectral domain, a periodic sound exhibits peaks in its spectrum at frequencies which are integer multiples (i.e. "harmonics") of a fundamental frequency (F0). The F0 corresponds to the inverse of the sound’s period. The pitch of a sound is most salient when a sound is composed of only harmonic frequencies, and the pitch height of such sounds corresponds to the F0. In many naturally occurring sounds, the F0 is the lowest harmonic present, but even if this frequency component is absent (in “missing fundamental” sounds), listeners can perceive a pitch at the highest common devisor of the sound’s remaining harmonics (Schouten, 1938). The brain can therefore use the relationship between harmonics, rather than the value of a physically-present F0, to compute pitch, and there is no direct relationship between the perceived pitch and the amount of sound energy at any one frequency.

**Fig. 1:**
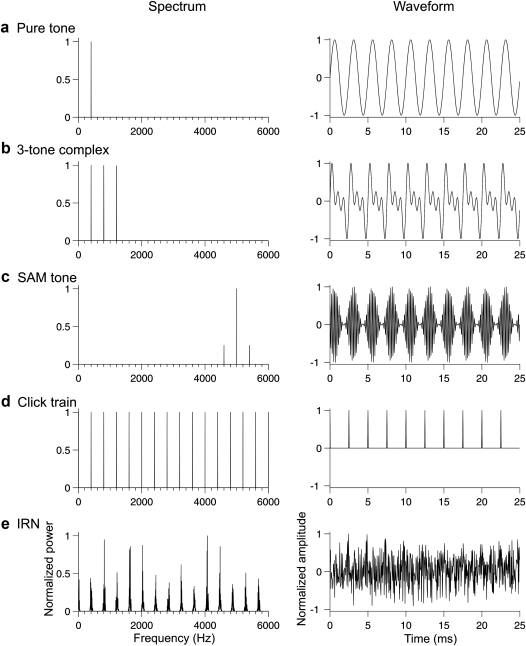
Some examples of periodic sounds. Each row shows the spectrum (left panel) and waveform (right panel) of a sound with an F0 of 400 Hz: (a) Pure tone; (b) Harmonic tone complex, containing tones at 400, 800 and 1200 Hz; (c) sinusoidally amplitude modulated (SAM) tone, with a carrier of 1000 Hz modulated by 400 Hz; (d) A train of clicks, with one click presented once every 2.5 ms; and (e) iterated rippled noise (IRN), with a delay period of 2.5 ms and 20 iterations.

Periodic sounds can take many forms. The pitch of a pure tone corresponds to its frequency (Fig. 1a). A tone complex containing frequencies that are harmonically related will be heard as a single auditory event with a pitch at F0, at least when enough low-order harmonics are present (Fig. 1b). When a pure tone is sinusoidally amplitude modulated (SAM), regions of “sideband” energy are created that flank the original pure tone “carrier” frequency at frequencies corresponding to: (a) the difference between the carrier and modulation frequencies, and (b) the sum of the carrier and modulation frequencies (Fig. 1c). If the carrier and modulation frequencies are harmonically related, then such a sound has a pitch corresponding to the modulation frequency. A periodic train of clicks (i.e. brief, broadband events) will have an associated pitch at the click repetition rate (Fig. 1d). This stimulus models the process that animals use to produce vocalizations at specific pitches: the controlled “clapping” of vocal folds at a desired rate. When a broadband noise is repeatedly delayed by a brief time period and added to the original signal, it becomes more temporally regular with each iteration of the delay-and-add process, although its waveform remains somewhat noisy (Fig. 1e). This stimulus is called iterated rippled noise (IRN), and it evokes a pitch corresponding to the inverse of the delay time. Pitch can even be evoked by a broadband noise that is aperiodic at each ear, but whose waveform is correlated across ears within a limited spectral band (Cramer and Huggins, 1958). This stimulus evokes a pitch (called “Huggins pitch”) within the region of interaural correlation, and it demonstrates that pitch can be extracted after the signals arriving at each ear are combined. The variety of pitch-evoking stimuli, which are often only approximately periodic and sometimes not at all, offers powerful tools for probing the neural mechanisms listeners use to compute pitch.

## 3 Do animals perceive pitch?

Sounds generated by animals are often periodic, and it is of great survival value for animals to form a robust representation of this acoustical feature. For example, the F0 of a resonant cavity is a function of both its volume and density, so a pitch-perceiving animal can tap a coconut to determine whether it is full or empty. Since pitch is defined as a perceptual attribute, it is difficult to demonstrate conclusively whether non-human animals experience pitch in a manner similar to us, but there is much evidence to suggest that they might.

For humans, pitch perception has not only made the development of music possible, but it also provides important vocal communication cues. The pitch of speech can inform listeners about the age or gender of a speaker (Gelfer and Mikos, 2005; Smith et al., 2005), as well as the emotional state of the speaker - excited speakers tense their vocal folds, thereby raising the F0 of their vocalizations (Fuller et al., 1992; Reissland et al., 2003). In tonal languages, or when a speaker uses intonation, F0 can change the meaning of a spoken word. Many animals generate vocalizations in an entirely analogous pulse-train-resonance fashion to humans, and these species similarly use periodicity and spectral cues to interpret vocalizations. Chimpanzees (*Pan troglodytes;* Kojima et al., 2003), rhesus monkeys (*Macaca mulatta*; Koda and Masataka, 2002), sparrows (*Spizella pusilla*; Nelson, 1989), and bullfrogs (*Rana catesbeiana*; Capranica, 1966) have been shown to identify con-specifics based on the periodicity of their vocalizations.

Animals exhibit the four properties of pitch perception outlined above (see Introduction). While human frequency and pitch discrimination thresholds are smaller than those of most other species (Shofner, 2005), animals can detect changes in the periodicity of complex sounds, including click trains (Marvit and Crawford, 2000), harmonic tone complexes (Dooling et al., 2002; Shofner, 2002), sinusoidally amplitude modulated noise bursts (Dooling and Searcy, 1981; Long and Clark, 1984; Moody, 1994), and iterated rippled noises (Fay et al., 1983; Shofner et al., 2007). Furthermore, animals can generalize their learning on a pitch discrimination task across very different stimulus types (Cynx and Shapiro, 1986; Heffner and Whitfield, 1976; Shofner, 2002). They are truly sensitive to periodicity, since rhesus monkeys (Tomlinson and Schwarz, 1988), European starlings (*Sturnus vulgaris*; Cynx and Shapiro, 1986) and cats (*Felis silvestris catus*; Heffner and Whitfield, 1976) can all be trained to respond to the pitch of missing fundamental sounds. Ferrets (*Mustela putorius furo*; Kalluri et al., 2008) and songbirds (*Taeniopygia guttata* and *Melopsittacus undulates*; Lohr and Dooling, 1998) can detect mistuned harmonics in tone complexes. Starlings (*S. vulgaris;* Hulse et al., 1995) and monkeys (*Macaca fuscata* and *M. mulatta*; Izumi, 2000; Wright et al., 2000) are also sensitive to the consonance and dissonance of complex sounds, and rhesus monkeys can generalize pitches across octaves to make pitch chroma judgments (Wright et al., 2000).

**Fig. 2:**
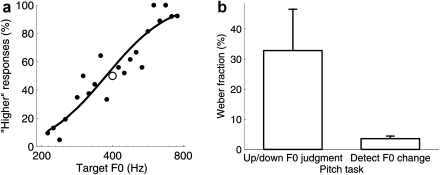
Ferrets’ discrimination performance on two F0 discrimination tasks. (a) Pitch direction judgment performance of one ferret on a two-alternative forced choice task (black dots). On each trial, a reference artificial vowel (F0 = 400 Hz, black open circle) was presented, followed by a second, target vowel of different F0 (*x*-axis). The ferret indicated, by water spout choice (*y*-axis), whether the target vowel was higher or lower in pitch than the reference. The black curve is a probit fit to the ferrets’ spout choices. (b) The mean (+standard deviation) of ferrets’ Weber fractions on two F0 discrimination tasks are shown. The Weber fractions of 4 ferrets were measured on the pitch direction judgment task described in (a), using references between 350 and 450 Hz (left; *n* = 6 thresholds). The Weber fractions of 3 further ferrets were measured on a go/no-go pitch change detection task (*right*). On each trial, the ferret was required to release a water spout when the F0 of a sequence of 400-Hz vowels changed. Detection of pitch increases and decreases were tested in separate sessions (*n* = 6 thresholds in total).

Monkeys (*Macaca mulatta,* *Cebus* *apella* and *M. fuscata*; Brosch et al., 2004; D’Amato, 1988; Izumi, 2001), rats (*Rattus norvegicus*; D’Amato, 1988) and birds (*S. vulgaris*, *T. guttata*, and *Columba livia*; Cynx, 1995; Page et al., 1989) can judge sequences of tones based on the direction of change in their pitch height, suggesting that they can judge the relative pitch of sounds on a low-to-high scale. However, these studies emphasize that animals are not easily trained to discriminate sounds based on their relative pitch. The animals preferred to judge periodic sounds according to their absolute pitch height. In our lab, we have trained ferrets on a task in which they hear two sequential artificial vowel sounds (i.e. formant-filtered click trains) and respond at one of two water spouts to indicate whether the second was higher or lower in pitch than the first (Fig. 2a). These experiments show that ferrets can respond to complex sounds based on the height of their periodicity along a high-to-low scale (Walker et al., 2009c). Since the reference was kept constant during a given testing session, the task could be solved by either responding to the direction of pitch change between the two vowels or the absolute pitch height of the second vowel. Probe trials in highly trained ferrets indicated that these animals adopted the latter of these strategies, attending to the absolute pitch of the target sounds. Therefore, ferrets, like rats, birds and monkeys, seem to attend more strongly to the absolute pitch of individual sounds than to relative pitch shifts.

Together, these experiments provide evidence that non-human animals are sensitive to many of the same periodicity cues as humans, with the caveat that animals may be less inclined to respond to the relative pitch of sequential sounds. Given the similarities in pitch perception across species, animal models may offer useful insights into common neural processes that give rise to pitch perception in a variety of species, including humans.

## 4 Subcortical representations of sound periodicity

Just as the acoustical correlates of pitch can be conceptualized in the temporal or spectral domains, theories of how the brain may extract pitch cues have also been based on temporal and spectral properties of neural responses. Temporal theories pose that pitch is computed from the *timing* of action potentials that are phase-locked to the sound waveform, while spectral theories suggest that pitch is represented as the *place* of activation across the tonotopic map (i.e. the anatomical organization of frequency selectivity). More recently, models of pitch extraction have tended to integrate spectral and temporal encoding principles (Carlyon, 1998; de Cheveigné, 2005).

Encoding the pitch of a pure tone is, in theory, relatively easy. For a given sound level, the pitch can be derived as the place of maximal activation along the tonotopic map as early in the nervous system as the auditory nerve. However, for complex sounds, there is no simple relation between pitch and frequency composition, and so more sophisticated neural computations are necessary to determine sound periodicity. Since auditory nerve fibres phase-lock to sounds, their firing patterns in response to a periodic sound are themselves periodic. A pure tone frequency can therefore be derived as the periodicity (or first-order autocorrelation) of spikes observed across responsive auditory nerve fibres. For complex sounds, F0 is encoded within the all-order autocorrelation of spikes across fibres (Cariani, 1999; Licklider, 1951; Meddis and O’Mard, 1997). Auditory nerve fibres phase-lock to frequencies up to about 5 kHz in the cat (Johnson, 1980), and this limit coincides closely with the upper limit of musical pitch perception in humans (Semal and Demany, 1990). In addition to this temporal signature of the sound waveform, the firing activity across the bank of tonotopically-arranged auditory nerve fibres provides an approximate spectrogram of the sound. The harmonic profile of a periodic sound, and thus the pitch, can be derived from this place code by the application of an appropriate spectral filter to the tonotopic map (Cohen et al., 1995; Goldstein, 1973), provided that the harmonics of the sound are spaced widely enough to produce resolved areas of activation. The limits of this place code, like the temporal code, also have psychophysical correlates. The strength of perceived pitch decreases for sounds that contain less resolved harmonics. However, listeners can still identify the pitch of sounds in which the harmonics are entirely unresolved (for pitches up to about 300 Hz), so in these cases temporal codes may be essential for pitch perception (Houstma and Smurzynski, 1990; Pierce, 1991; Shackleton and Carlyon, 1994).

Auditory nerve fibres synapse onto cells in the cochlear nucleus in the brainstem. The cochlear nucleus contains more than 20 cell types, which are distinguishable based on their morphologies, response properties, and projection targets (Brawer et al., 1974; Pfeiffer, 1966). The functional role of many of these neuron types is not yet known, but some exhibit firing properties that are ideally suited to process sound periodicity. Primary-like cells have similar response properties to auditory nerve fibres, and so they preserve information about the fine temporal structure of sounds that might be required for pitch extraction by higher brain areas (Winter and Palmer, 1990). Chopper neurons respond to periodic inputs with temporally precise, periodically occuring spikes, and the probability of firing in these units is proportional to the number of synchronous inputs they receive from auditory nerve fibres. These cells represent the F0 of a complex sound as the reciprocal of their inter-spike intervals, thereby converting the all-order inter-spike interval code of stimulus periodicity in auditory nerve fibres into a first-order inter-spike interval code (Winter, 2005). A functional Magnetic Resonance Imaging (fMRI) study of the human auditory system has shown that metabolic rate of the cochlear nucleus is dependent upon the temporal regularity of sounds (Griffiths et al., 2001). Although the temporal firing patterns, but not overall firing rates, of onset chopper neurons have been shown to depend on sound periodicity, mathematical models have suggested that the synchronization of firing across neurons may lead to an overall increase in firing rate that could be measurable with fMRI (Chawla et al., 1999).

In the next station of the classical auditory pathway, the superior olive, sound signals from both ears are first combined. The existence of binaural pitch suggests that mechanisms for pitch extraction exist at or beyond this level of the auditory system (Cramer and Huggins, 1958). Neurons in the superior olive project to the inferior colliculus, where the spectral features of sounds are further processed via lateral inhibition across the tonotopic map. This may serve to sharpen the neural representation of the spectral peaks of complex sounds, aiding template-matching (i.e. place code) approaches to pitch extraction (McLachlan, 2009). Similar mechanisms have also been observed in the cochlear nucleus (Rhode and Greenberg, 1994). Many neurons in the inferior colliculus show band-pass sensitivity to the frequency of sinusoidal amplitude modulation of sounds (Rees and Møller, 1987). This sensitivity is evident in phase-locked responses to stimuli with modulations of up to 500 Hz, but is encoded with unsynchronized spike rates for faster modulations, up to 1 kHz (Langner and Schreiner, 1988). Using SAM tones as stimuli, Langner et al. (2002) have suggested that a “periodotopic” map of best modulation frequency exists in the inferior colliculus, running orthogonal to the direction of the tonotopic map. This would constitute the earliest map of periodicity present in the auditory system, although the results of McAlpine (2004) suggest that these organized responses may be explained by the presence of cochlear distortion products rather than periodicity encoding (further discussed in Section 5.1). Further evidence for a contribution of the inferior colliculus to the representation of periodicity is provided by the finding that, in humans, the blood oxygenation response to periodic sounds in the cochlear nucleus is enhanced in the inferior colliculus (Griffiths et al., 2001).

## 5 Pitch processing in auditory cortex

### 5.1 Representations of sound periodicity in auditory cortex

While neurons in subcortical nuclei of the auditory pathway encode periodicity cues, lesion studies suggest that auditory cortical function is necessary for pitch perception. In mammalian species, the auditory cortex consists of several fields, which are distinctive in terms of their physiological responses to sound (Bizley et al., 2005; Downman et al., 1960; Merzenich and Brugge, 1973; Thomas et al., 1993), and their anatomical connections (Bajo et al., 2007; Budinger et al., 2000; Hackett, 2008; Winer and Lee, 2007). The functional organization of the higher cortical fields is poorly understood, but these regions appear to divide into anatomically segregated processing streams that process sound features for distinct behavioural purposes (Lomber and Malhotra, 2008; Rauschecker et al., 1997; Romanski et al., 1999). In humans, the primary and a secondary auditory cortical field are found on an anatomical landmark known as Heschl’s gyrus (HG), and higher centres of auditory cortex can be found both anteriorly, on the planum polare, and posteriorly, on the planum temporale. The roles of these cortical regions in pitch processing have been investigated using a variety of techniques, but two very fundamental questions continue to be debated. Firstly, whether ordered representations of pitch (“periodotopic” maps) exist in auditory cortex, and secondly, whether cortical representations of pitch are present in primary auditory cortex or arise from the specialized processing of higher cortical fields.

The frequency of pure tones is represented as a place code along the tonotopic map of primary auditory cortex (A1), as well as some secondary cortical fields. Some reports have suggested that, in addition to its tonotopic map, A1 may also feature a periodotopic arrangement. Pantev et al. (1989), using magnetoencephalography (MEG), found that the same regions of A1 were activated by pure tones and missing fundamental tone complexes that were of the same F0. They concluded that the tonotopic map of A1 is actually a periodotopic one. In contrast to this result, Langner et al. (1997) found that MEG responses in auditory cortex showed the expected topographic arrangement for pure tones, but an orthogonal periodotopic map for the pitch of missing fundamental tone complexes. The discrepancy in these results may be explained by differences in the use of stimulus controls. Missing fundamental sounds are known to produce a mechanical artefact in the cochlea, which is centred at the F0 within the tonotopic map of the basilar membrane. Neural responses to this F0 artefact could result in tonotopic activation in A1. Such responses could also manifest as activation along the maps of sound bandwidth or intensity that have been proposed to lie orthogonal to the A1 tonotopic gradient (e.g. Heil et al., 1992). Pantev et al. (1989) aimed to control for this artefact by including a noise band centred at the F0 within their tone complex. However, because this noise band was as intense as the tone complex itself, the neural response to the sound’s periodicity may be simply accounted for as a response to the noise. The presentation of a similar noise band at an intensity that was sufficiently low so as to not evoke the MEG response would have controlled for this possible confound. On the other hand, Langner et al. (1997) did not include a control for cochlear distortion products. While this may account for why their results are different from those of Pantev et al. it similarly leaves room for doubt about whether the responses they observe result from the sound periodicity or cochlear artefacts. The same caveat may be raised about the demonstrations of periodotopic maps in A1 of the Mongolian gerbil (*Meriones unguiculatus*), where SAM tones where presented without controls for cochlear distortion products (Schulze et al., 2002). The relevance of orthogonal representations of periodicity and frequency to pitch perception is not immediately obvious. One might expect that if a map of stimulus periodicity exists in A1, it should be independent of sound spectrum and thus lie along the tonotopic map, since pure tones have a well-defined periodicity and evoke a pitch that is comparable to that of their tone complex counterparts. Thus, while the idea of a periodotopic map in auditory cortex is appealing, the experimental evidence for such an arrangement, and its contribution to pitch perception, remains inconclusive.

Patterson et al. (2002) used fMRI to distinguish among the relative contributions of auditory cortical fields to pitch processing. They measured the blood oxygenation response in human listeners during the presentation of iterated rippled noise and broadband noise bursts, and found that only a select region of auditory cortex, the lateral HG, was more strongly activated by pitch-evoking stimuli than by the aperiodic noises. In support of this result, Penagos et al. (2004) found that fMRI activation in lateral HG correlated with pitch salience – that is, the number of resolved harmonics present in tone complexes. Gutschalk et al. (2002) measured the magnetic field associated with the presentation of regular and irregular click trains, and found that the activity of a current source located in lateral HG was dependent on the temporal regularity of the stimulus, but not sound level. Moreover, this activity was only associated with periodic sounds when they were presented above the lower pitch limit. Schönwiesner and Zatorre (2008) were able to study the function of lateral HG more directly by recording local field potentials with depth electrodes that were implanted into the superior temporal lobe of a patient in preparation for surgical treatment of epilepsy. They showed that the presentation of iterated rippled noises elicited a stronger response in the lateral portion of HG than aperiodic noises, while the opposite result was found in medial HG. Furthermore, only the lateral portion of the superior temporal plane was sensitive to the onset of IRN within continuous noise.

These studies suggest that a particular region of auditory cortex is specialized for processing pitch. However, to confirm and extend such a conclusion it is necessary to discover how single neurons within auditory cortex encode stimulus periodicity in their spike responses and to show that those responses are correlated with behavioural measurements of pitch perception. While subcortical stations may represent the periodicity of pitch-evoking sounds within their inter-spike intervals, auditory cortical neurons are less able to synchronize their firing to fast rates of modulation (Wang et al., 2008). Instead, sound periodicities within the pitch range are more likely to take the form of spike rate modulations or more sparse timing codes at the level of auditory cortex. Click trains with repetition rates up to about 300 Hz are represented isomorphically in the phase-locked discharges of a subset of A1 neurons in awake Macaque monkeys, while a separate group of A1 neurons use monotonic increases in spike rate to represent faster repetitions (Steinschneider et al., 1998). This upper limit of phase locking in A1 is near the upper limit of temporally-based pitch perception for unresolved harmonics (Pierce, 1991). Lu et al. (2001) studied spiking responses to click trains in A1 of awake marmoset monkeys (*Callithrix jacchus*), and they found a similar combination of synchronized and spike rate representations of click train periodicity in separate neural populations. They observed that monotonic spike rate representations were preferred over synchronized responses for click rates beyond about 40 Hz, which is near the lower limit of pitch perception in humans (Krumbholz et al., 2000). The representation of periodicities in the pitch range in the form of both spike rate and temporal pattern codes seems to occur, at least in part, already in the auditory midbrain (Langner and Schreiner, 1988; Schreiner and Langner, 1988). The synaptic mechanisms underlying the conversion of an explicit temporal encoding of stimulus periodicity to a spike rate code have not yet been identified.

A1 neurons are also sensitive to spectral cues of sound periodicity, since some of the neurons found there respond to harmonics of their characteristic frequency (Kadia and Wang, 2003; Qin et al., 2005; Sutter and Schreiner, 1991). These neurons not only respond to these harmonics when they are presented in isolation, but can also show an enhanced response to their characteristic frequency in the presence of harmonics. These neurons would be ideal candidates for template-matching theories of pitch extraction, but the characteristic frequencies of those neurons identified so far lie outside the pitch range (> 5 kHz). A small proportion (˜12%) of neurons in A1 and in the anterior auditory field of the ferret have spectrotemporal receptive fields with multi-peaked frequency tuning properties that reliably distinguish between harmonic and inharmonic tone complexes (Kalluri et al., 2008).

The findings described so far point to an important role for the spike rates of auditory cortical neurons in representing pitch. Since pitch sensitivity in the human lateral Heschl’s gyrus is evident with fMRI, MEG and measurements of local field potentials, it appears to take the form of a net modulation of firing activity in large neural populations, across relatively wide time periods. Single unit studies have demonstrated that primary auditory cortical neurons are equipped to represent both temporal and spectral periodicity cues, although unlike in brainstem structures, these cues are predominantly represented as spike rate modulations rather than phase-locked temporal discharge patterns. Many neurons throughout A1 exhibit spike responses that are modulated by the periodicity of complex sounds, and while there has been some evidence for periodotopic responses to missing fundamental sounds, the majority of studies have failed to demonstrate a topographic arrangement of the periodicity preferences of cortical cells (e.g. Bendor and Wang, 2005; Cansino et al., 2003; Fishman et al., 1998; Nelken et al., 2008; Schwarz and Tomlinson, 1990).

### 5.2 Cortical correlates of pitch change detection

We have seen that activity in Heschl’s gyrus of the human auditory cortex is sensitive to the temporal regularity of complex sounds, and we now ask how this sensitivity may contribute to listeners’ ability to detect changes in pitch height. Neurological patients with bilateral damage to HG are impaired in detecting changes in the frequency of pure tones (Kazui et al., 1990; Tramo et al., 2002), as well as pitch changes in tone complexes and missing fundamental sounds (Tramo et al., 2004). Bilateral ablation of auditory cortex in rhesus monkeys (Harrington et al., 2001) and bilateral inactivation of A1 in rats (Talwar et al., 2001b) have also been shown to impair the performance of these animals on tasks in which they must respond to a frequency change within a pure tone sequence.

In humans, right auditory cortical infarctions are more likely to impair pitch discriminations than damage to the left hemisphere (Divenyi and Robinson, 1989; Robin et al., 1990; Sidtis and Volpe, 1988; Stewart et al., 2006). Furthermore, fMRI activation in the right planum temporale of healthy listeners is correlated with the size of frequency shifts presented between successive tones, but this correlation is not present for left auditory cortical activity (Hyde et al., 2008). This result may be interpreted to suggest that the right auditory cortex has finer spectral resolution than the left, and that pitch discrimination function is at least partially lateralized. Hemispheric specialization for the detection of pitch changes in sequential tones has not been documented in non-human animals, and so this form of lateralization of pitch function may be especially pronounced in humans.

The cortical correlates of pitch change detection have been examined using MEG. One line of research has focussed on the “pitch onset response”, which is evoked in response to a change in the pitch of an ongoing periodic sound, or to the onset of pitch in continuous noise. The latency of the pitch onset response is determined by the pitch height, and the amplitude is proportional to the pitch salience (Krumbholz et al., 2003; Ritter et al., 2005). Although the pitch onset response is transient, it has a longer latency than the response to the onset of sounds in silence, which is consistent with this neural activity resulting from pitch computations that are calculated over several cycles of the fundamental period. The source of the pitch onset response is thought to reside in lateral HG for transitions between IRNs that differ in pitch (Ritter et al., 2005). It has been attributed to a slightly more medial source in lateral HG for transitions from noise to pitch-evoking IRN (Krumbholz et al., 2003). This may suggest that the anatomical lay-out of the processes used to detect the onset of periodicity are different from those used to detect a pitch change, but note that it is impossible to determine the precise location of MEG sources with absolute certainty (Wendel et al., 2009). The sources of MEG responses are localized by solving a mathematical inverse model to account for the results, but there are always multiple alternative solutions that are consistent with the data. While the source locations of MEG studies must be considered with this caveat in mind, the attribution of the pitch onset response to lateral HG is supported by independent results of fMRI (Patterson et al., 2002) and depth electrode (Schönwiesner and Zatorre, 2008) studies, which more directly measure the spatial distribution of neural responses to pitch changes.

While the monotonic spike rate/periodicity functions of A1 neurons described in Section 5.1 may be able to account for listeners’ detection of pitch changes, this has not yet been studied in detail. Preliminary results from our lab suggest that the spike rate responses of ferret auditory cortical neurons are sufficient to support ferrets’ pitch change detection thresholds for artificial vowel sounds (Walker et al., 2009a). There has, however, been much research into the changes in frequency receptive field properties of auditory cortical neurons that result from performing frequency change detection tasks. The association of a pure tone frequency with an aversive stimulus in classical conditioning studies (Bakin and Weinberger, 1990; Blake et al., 2006; Edeline and Weinberger, 1993; Galvan and Weinberger, 2002), or the trained response to a change in pure tone frequency on an operant conditioning task (Blake et al., 2002; Fritz et al., 2003), results in an enhancement in auditory cortical neurons’ response to the conditioned frequency. This effect is usually accompanied by decrease in responsiveness to an unconditioned tone, as well as other untrained frequencies (Blake et al., 2002; Edeline and Weinberger, 1993; Fritz et al., 2003; Galvan and Weinberger, 2002). This selective pattern of inhibition and excitation provides a spectral sharpening of the relevant frequency contrast (Ohl and Scheich, 1997), which can occur over very rapid time scales, and can last for hours (Fritz et al., 2005). Spectral sharpening can also occur in these paradigms in the form of an overall decrease in response to sounds, with a relative increase in firing rate in response to the test frequency (Witte and Kipke, 2005).

Frequency discrimination studies in animals have also reported an enlargement in the area of representation of the test frequency within the tonotopic map of A1, up to 9 times that of control animals (Polley et al., 2006; Recanzone et al., 1993; Rutkowski and Weinberger, 2005). This process is thought to be mediated, in part, by inputs to auditory cortex from the nucleus basalis (Kilgard and Merzenich, 1998). However, at least one other study has failed to find tonotopic map reorganization in cats following frequency discrimination training (Brown et al., 2004), so this form of plasticity may be highly dependent on the association between reward and presentation of test stimuli in the experimental paradigm. Talwar and Gerstein (2001) artificially induced an enlargement in the representation of certain frequencies in the A1 tonotopic map using intracortical microstimulation, and this form of map reorganization did not affect frequency discrimination behaviour in the rat. Therefore, an increase in the tonotopic representation of a frequency band alone is not sufficient to induce perceptual learning on change detection tasks. To further complicate matters, Recanzone et al. (1993) observed an increase in the latency of A1 neuron responses to the test frequency following discrimination training in owl monkeys (*Aotus azarae*), while Brown et al. (2004) found that cat A1 neurons had shorter latency responses to test stimuli resulting from such training. Clearly, many questions remain about how cortical plasticity may underlie learning on tasks that require the discrimination of pure tone frequencies. In comparison, even less is known about the cortical mechanisms that allow us to detect fine changes in the pitch of complex sounds.

### 5.3 Representations of the direction of pitch shifts in auditory cortex

The ability to perceive the pitch of periodic sounds along a continuous scale, from low to high, can allow a listener to estimate continuous properties of the sound source, such as large or small, relaxed or tense, empty or full. A high/low pitch classification is not equivalent with the mere detection of a pitch change. For very small pitch shifts, listeners can sometimes detect that the periodicity of a sound has changed, but are unable to determine whether the pitch increased or decreased. This effect has been demonstrated in children with cochlear implants (Vongpaisal et al., 2006), as well as healthy, adult listeners (Semal and Demany, 2006). This effect has also, somewhat counter-intuitively, been found to be reversed in listeners with superior pitch discrimination thresholds (Semal and Demany, 2006). We have observed that ferrets can detect a change in the F0 of a train of artificial vowel sounds at very fine resolutions, while their F0 difference thresholds are much higher when they are required to judge the direction of pitch change between these sounds (Fig. 2b; Wilcoxon rank-sum test; *p* < 0.01). We are currently investigating the auditory cortical correlates of ferrets’ performance across these tasks (Walker et al., 2009a).

The results of auditory cortical damage in human neurological patients suggest that pitch change detection and direction judgments involve, to some extent, anatomically distinguishable neural processes. Tramo et al. (2002) have shown that while control listeners show similar thresholds on a frequency change detection and direction discrimination task, bilateral damage to auditory cortex does not result in similar degrees of impairment on these tasks. Rather, a patient with bilateral auditory cortical infarcts produced thresholds on the direction discrimination task that were about twice as large as those for frequency change detection. Similarly, an impairment in pure tone frequency direction judgments, but not frequency change detection thresholds, has been observed in patients with surgical lesions of the right temporal gyrus that included lateral HG (Johnsrude et al., 2000). Pitch discrimination performance was within normal limits on both these tasks for patients with auditory cortical lesions that included only left hemisphere structures or for those in which the lateral portion of HG was spared. Tramo and colleagues have also reported elevated frequency direction judgment thresholds in a patient with damage to the right temporal, but not superior temporal, gyrus (Tramo et al., 2005). In a thorough review of studies that examine pitch perception in neurological patients, Stewart et al. (2006) show that impairments on both these pitch tasks are often found in patients with damage to lateral HG, planum temporal, and the parieto-temporal junction, especially in the right hemisphere. These authors also suggested that pitch difference detection is associated with subcortical structures and primary auditory cortex, whereas pitch direction discrimination is more often associated with lateral HG.

Another form of lateralization for pitch direction judgments has been highlighted in healthy listeners, using MEG and structural MRI (Schneider et al., 2005). As in the neurological studies above, subjects were asked to report the direction of a pitch change between two successive sounds. But here, instead of pure tones, tone complexes with missing fundamentals were used as stimuli. The stimuli were designed such that if listeners derived pitch purely from the spectral content of the sound (i.e., the lowest harmonic present), they perceived a pitch shift of opposite direction than if they responded to the missing fundamental pitch (i.e., based on harmonic spacing). Listeners who made missing fundamental judgments had stronger MEG responses to pitch shifts in the left HG, and MRIs showed that these individuals also had a greater volume of grey matter in left lateral HG than in the right hemisphere. The opposite lateralization of HG activation and grey matter volume was found in subjects who used a spectral pitch strategy. This suggests that the impairment in pitch direction judgments reported in neurological patients with right temporal lobe damage may result from an inability to analyse the spectral content of sounds. However, this interpretation is at odds with the results of Zatorre (1988), who found that right, but not left, HG lesions impair missing fundamental judgments.

Together, examinations of neurological patients seem to suggest that while the effects of right and left auditory cortex damage on frequency discrimination tasks are additive (that is, the most profound deficits result from bilateral damage), the right non-primary auditory cortex often plays a necessary role in pitch direction judgments. The involvement of secondary auditory cortex lesions is in general agreement with the results of fMRI (Patterson et al., 2002; Penagos et al., 2004) and MEG (Gutschalk et al., 2002) studies, which proposed that lateral HG makes a unique contribution to pitch processing. However, a right hemisphere dominance for pitch processing at the level of lateral HG has not been observed in fMRI investigations of healthy listeners (Patterson et al., 2002; Penagos et al., 2004). Perhaps both hemispheres contribute to pitch judgments in the healthy human brain, but the role of the right hemisphere may be a more necessary (i.e. more unique) one. Additionally, there may be a bias towards finding pitch impairments in individuals with right hemisphere lesions, as damage to the left temporal cortex often results in language impairments that make musical testing difficult and low-priority (as suggested by Stewart et al., 2006). Along similar lines, surgical lesions of temporal cortex for the treatment of epilepsy tend to be more restricted in the left hemisphere, due to the danger of impairing language function on this side of the brain.

In non-humans, missing fundamental perception has not been found to be lateralized. Bilateral, but not unilateral, lesions of auditory cortex in cats impair their ability to judge the direction of pitch shifts in tone complexes with missing fundamentals (Whitfield, 1980). Cats retain the ability to perform this pitch direction task using pure tones or tone complexes that include F0. Studies of frequency-modulated tone discrimination in gerbils (*M. unguiculatus*) and rats have suggested that the right auditory cortex of these animals may play a greater role in spectral judgments and the left in processing temporal cues (Rybalko et al., 2006; Wetzel et al., 1998, 2008). This is consistent with the lateralization of function observed in humans, but the differential roles of right and left auditory cortex in processing the pitch of complex sounds has not yet been directly demonstrated in animal models, as most extracellular recording studies are carried out in only one hemisphere.

Higher auditory cortical regions may also be functionally specialized for different pitch tasks. Warren et al. (2003b) used fMRI to measure cortical responses while human subjects passively listened to a series of sounds that varied in pitch. Using subtraction techniques, they identified an area posterior to HG, in the planum temporale, that responded selectively to changes in the height, but not the chroma, of tone complexes. Therefore, while we have seen that lateral HG may play a key role in processing the periodicity of sounds, areas of auditory cortex beyond this region may be specialized for processing different aspects of pitch, or for applying pitch cues to different functional purposes. The functional lateralization of pitch processing may continue beyond auditory cortical fields. An fMRI study of healthy listeners has demonstrated that discriminating the direction of pitch changes in speech sounds is associated with selective activation of right prefrontal cortex regions (Zatorre et al., 1992).

Given the dissociation between listeners’ ability to order and discriminate pitch differences, we might expect to find separate neural underpinnings for these judgments at the single neuron level. Brosch and colleagues recorded from primary and secondary auditory cortical neurons in rhesus monkeys, and showed that the first tone in a two-tone sequence can inhibit or enhance the spike rate response to the second tone (Brosch and Scheich, 2008; Brosch et al., 1999). The response of any one neuron was often enhanced specifically by either frequency increases or decreases, so that these cells functioned as frequency shift detectors. However, the frequency of the second tone was kept constant for each neuron, so it is not yet known if cortical neurons can respond to a direction of pitch change independently of the absolute frequency of the tones. The same group has also trained monkeys to respond to downward, but not upward, frequency shifts in tone sequences (Brosch et al., 2004). Neural responses were recorded in A1 and the caudomedial belt while the monkeys performed this task, and two classes of informative neural responses were identified (Selezneva et al., 2006). Neurons exhibiting phasic responses to the tones reliably represented the direction of frequency shifts presented, while other tonically-responsive neurons had firing rates that correlated with monkeys’ behavioural choices on the task. This important work demonstrates that neurons in early auditory cortical stations represent both stimulus parameters and perceptual decisions. In a similar experiment, Yin et al. (2008) trained rhesus monkeys to identify a 4-tone sequence. They also observed both stimulus-specific modulations of responses to tones, and responses that were time-locked to, and predictive of, the monkey’s behavioural response. Both response types were found among A1 neurons, but were more common within a secondary auditory cortex region (field R).

We have also investigated the relations between cortical representations of periodicity and animals’ pitch height judgments. In our experiments, we used complex sounds, rather than pure tones, so that the task could not be solved by simply attending to the maximal place of activation along a tonotopic map. As described earlier (Section 3), ferrets were presented with two sequential artificial vowel sounds on each trial, and were required to indicate, by water spout choice, whether the second sound was higher or lower in F0 than the first (Walker et al., 2009c). In separate neurophysiological studies carried out in untrained, anaesthetized ferrets, we found that the F0 of these artificial vowels modulated the spike rate responses of a subset of cortical units (i.e. single neurons and clusters of small numbers of neurons) that were distributed through five fields of the left auditory cortex in ferrets, including both primary and secondary regions (Bizley et al., 2010). Approximately 38% of neurons that were sensitive to vowel F0 showed monotonically increasing rates of firing across the range of F0 tested (“high-pass” neurons), while another 38% of neurons decreased their firing rate with increasing F0 (“low-pass” neurons).

**Fig. 3:**
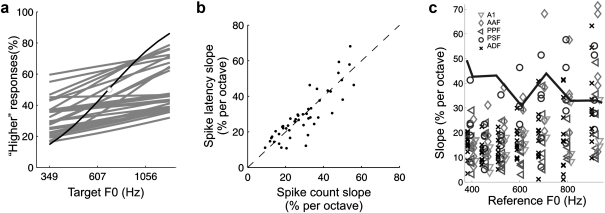
Neurometric analysis of how well monotonic auditory cortical codes of artificial vowel F0 support pitch discrimination judgments (modified from Bizley et al., 2010). (a) Neurometric “F0 discrimination” curves for a population of 26 auditory cortical neurons that were simultaneously recorded in an anaesthetized ferret. The light gray curves show neurometrics for each individual neuron, while the black curve shows the neurometric performance based on the response of the population of 26 cells. The white circle indicates the reference value. (b) The scatter plot compares the slopes of neurometric curves for populations of auditory cortical neurons when calculated using either the number of spikes (*x*-axis) or relative first-spike latency (*y*-axis) as a response. (c) A comparison of ferrets’ psychometric and auditory cortical neurometric sensitivity. The black line shows the mean psychometric slopes of ferrets on a two-alternative forced choice pitch direction judgment task, across a range of reference F0s. The symbols plot the neurometric slopes for populations of auditory cortical neurons. Different symbols are used for populations from different cortical areas, as shown on the legend.

To investigate the potential behavioural significance of this distributed representation of vowel periodicity, we used neurometric analyses to determine whether this monotonic spike rate representation was sufficiently reliable to provide the physiological signal upon which ferrets made their behavioural judgments. These analyses described performance on our pitch discrimination task by an observer of a neurons’ activity. If the neuron was high-pass, the observer made “higher” and “lower” pitch judgments on each trial based on whether the neuron’s firing rate in response to the target was higher or lower than in response to the reference, respectively (and vice versa for low-pass neurons). The spike rates of individual neurons were rarely able to account for the discrimination performance of ferrets, but the responses of small ensembles of neurons (comprising 3–61 simultaneously recorded neurons), when analyzed with a simple classifier, often discriminated periodicity changes as well as ferrets (Fig. 3a). The response of a neural ensemble was represented as the vector of spike rate responses of individual neurons in the ensemble. Each ensemble response was then classified according to whether it more closely resembled (i.e. was smaller in Euclidean distance to) the ensembles’ average response to a high F0 or a low F0 target. Compared to single neurons, the neurometric performance of neural ensembles was much more robust across a wide range of reference periodicities and sound levels. Codes based on either the relative first-spike latency or spike count provided neurometric curves that reached ferret behavioural thresholds (Fig. 3b). Highly sensitive ensembles were particularly common in the anterior primary auditory field and a posterior secondary field of auditory cortex, but neurometrics capable of matching psychoacoustic performance were found in all five cortical areas. Therefore, it appears that this particular form of periodicity representation is not limited to a specialized pitch centre (Fig. 3c).

In summary, further research is necessary to clarify whether or to what extent judging the direction of pitch shifts is a faculty that is lateralized or localized to specialized cortical regions. On the whole, human neurological, MEG and functional imaging studies provide compelling evidence that at least some pitch functions are lateralized to the right hemisphere and are carried out within certain higher auditory cortical centers (namely, lateral HG and planum temporal). However, the lateralization of function at the level of HG is not always clear. In gerbils, the discrimination of upward versus downward frequency modulations of continuous sounds is also lateralized to the right hemisphere. On the other hand, as these animals show no deficit following unilateral auditory cortex lesions when trained to discriminate frequency modulated sounds that are segmented (Wetzel et al., 2008), it is unclear how their right auditory cortex function may relate to the lateralization seen in humans for tasks in which subjects must judge the direction of pitch shifts in discrete, sequential sounds (Johnsrude et al., 2000; Tramo et al., 2002; Schneider et al., 2005). Pitch function may also be less specialized across cortical fields in non-primates. In ferrets, neurons that carry information about the F0 height of sounds, and which can account for the pitch direction discrimination thresholds of these animals, can be found throughout primary as well as higher auditory cortical fields. Nevertheless, the role of these neural populations in pitch discrimination performance has not yet been directly tested, so it remains possible that even these animals will show pitch discrimination deficits following inaction of specific cortical fields. Bendor and Wang (2008) have suggested that the subset of auditory cortical neurons that have monotonic spike rate/F0 functions may be particularly advantageous for making high/low pitch comparisons, while the non-monotonic rate codes of other auditory cortical neurons may contribute more effectively to detecting a change in F0. This intriguing hypothesis warrants further investigation, ideally in awake, behaving animals.

### 5.4 Invariant representations of pitch in auditory cortex

Humans and animals alike can generalize pitches across sounds with very different timbres. That is, a violin or a bird can produce sounds that evoke the common pitch percept of 800 Hz. One might thus expect to find auditory cortical neurons that encode the pitch of sounds independent of other sound features, such as timbre and loudness. This would require neurons with receptive field properties that go beyond simple frequency tuning. One can test for invariant pitch responses by examining cortical correlates of periodicity across a range of stimuli, including those that almost never occur in nature, such as pure tones or stimuli that evoke Huggins pitch. We might also expect the response of an ideal pitch-selective neuron to vary with pitch salience, and this can be tested with stimuli such as irregular (“jittered”) click trains or sounds with unresolved harmonics.

In contrast to the prediction of the existence of an invariant pitch representation, the cortical correlates of pitch perception have most often been found to vary with the type of stimulus presented, and thus the type of computations required by neurons to calculate F0. For example, while MEG investigations have localized the pitch onset response associated with binaural Huggins pitch stimuli in Heschl’s gyrus (Chait et al., 2006; Hertrich et al., 2005), an fMRI study has suggested that the neural correlates of Huggins pitch exist not in lateral HG, but rather in planum temporale (Hall and Plack, 2007). Hall and colleagues further show that the presentation of different types of periodic sounds, (including pure tones, resolved and unresolved tone complexes, Huggins stimuli, and IRN), activates different regions of auditory cortex in human listeners (Hall and Plack, 2009). In agreement with previous studies, they found that IRN stimuli evoked greater activity in lateral HG than did noise bursts. The novel finding of Hall and Plack (2009) was that other types of periodic sounds do not selectively activate this region. Instead, each stimulus resulted in a unique distribution of cortical activity, with the only area of overlap being planum temporale. Taking a similar experimental approach, Nelken et al. (2008) measured the intrinsic optical signals of primary and secondary regions of ferret auditory cortex while presenting click trains, SAM tones and iterated rippled noises across a common F0 range. The three types of periodic sounds resulted in three distinct patterns of periodotopic activation spanning several auditory cortical fields, but there was no consistent overlap in these F0 representations. Using the same methods, in addition to extracellular recordings, Langner et al. (2009) found that while harmonic tone complexes and SAM tones with the same periodicity often maximally activated similar regions of A1, pure tones with the same periodicity did not share this representation.

In contrast, Puschmann et al. (2010) have found, using fMRI, that the presentation of pitch sequences in the form of two types of dichotic pitch or pure tones in noise results in selective activation of lateral HG. One key difference between Puschmann et al. (2010) and the studies by Hall and Plack (2007, 2009) and Nelken et al. (2008) is that in the former, subjects carried out an auditory discrimination task during the stimulus presentation and image acquisition. They were asked to indicate, by keypress, whether each sound sequence consisted of noise, a fixed pitch, or a melody. In the studies by Hall and Plack (2007, 2009), subjects were asked to attend to the pitch of sounds but did not perform a behavioural task. The task-dependent plasticity results reviewed above indicate that perceptual tasks can have significant effects on the receptive field properties of cortical neurons. Therefore, differences in the perceptual task could alter the observed sensitivity of neurons in particular cortical fields to stimulus attributes such as pitch. For instance, lateral HG neurons might be differentially recruited when a subject is asked to isolate periodic sounds in the presence of a noisy background – an unavoidable function for all three periodic stimuli used in Puschmann et al.’s experiment. Future studies which more closely examine the task-dependent nature of neural correlates of periodicity may provide a coherent account of these seemingly contrary experimental results. For now, the bulk of evidence has failed to isolate a universal “pitch centre” within the auditory cortex of humans or animals, in which neurons represent pitch invariantly to the spectral make-up of sounds.

In natural acoustic environments, sounds do not vary over only one perceptual dimension independently (as they do in most experiments), but pitch changes must be recognized in the presence of other forms of stimulus variance. For instance, a speaker may move around the room, requiring the listener to de-convolve neural responses to pitch shifts from neural modulation by spatial location cues. We have recently examined how ferret auditory cortical neurons encode multiple perceptual dimensions by recording the neural responses to a stimulus set that varied simultaneously in F0, timbre and spatial location (Bizley et al., 2009b). We again used artificial vowels as stimuli, which allowed us to parametrically vary stimuli across four timbres (formants corresponding to the vowels/a/,/i/,/ɛ/, and/u/), F0 (click rates of 200, 336, 565, and 951 Hz), and spatial location along the azimuth (−45°, −15°, +15°, and +45°, where negative values are contralateral to the recording site). The parameters chosen for each perceptual attributes are easily discriminated by ferrets (Bizley et al., 2009a; Parsons et al., 1999; Walker et al., 2009c). We quantified neurons’ sensitivity to each of these three features using a variance decomposition analysis, based on multivariate ANOVA. Neurons that were sensitive to F0, timbre and azimuth were found in all 5 cortical fields examined. In fact, neurons were commonly modulated by two or more of these stimulus features (65%). Those that were tuned to only one stimulus dimension were less common (23%) and often tuned to the timbre dimension. Therefore, if invariant responses to periodicity exist within these cortical fields, they are rare. Sensitivity to the pitch and spatial location of stimuli has also been shown to have overlapping distributions in auditory cortex in fMRI, MEG and electroencephalographic investigations of human listeners (Degerman et al., 2008; Staeren et al., 2009).

**Fig. 4:**
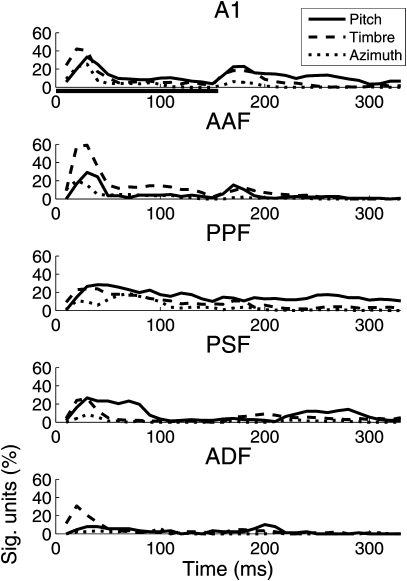
Proportions of cortical neurons modulated by the pitch, timbre or azimuth of complex sounds. In each panel, the proportion of ferret auditory cortical neurons with spike rates that are significantly modulated by the pitch (solid line), timbre (dashed line), or azimuth location (dotted line) of artificial vowels is indicated. Mutual Information was calculated for spike counts within 20 ms time bins, across the duration of the response. The significance of mutual information was determined using the 95% confidence interval of bootstrapped, “scrambled” responses (as described in Panzeri et al., 2007). This is compared in 20 ms time bins, across the duration of the response. The five panels, from top to bottom, show sensitivity across five cortical fields: A1 (primary auditory cortex), AAF (the Anterior Auditory Field), PPF (the Posterior Pseudosylvian Field), PSF (the Posterior Suprasylvian Field), and ADF (the Anterior Dorsal Field).

Another important result of our study was that a single spike count measure did not capture much of the informative variance in the responses of these neurons. Previous studies have usually looked for invariant pitch codes in the form of spike rates that are modulated selectivity by the periodicity of sounds. However, spike trains are often temporally complex, and different aspects of a neural spiking pattern can be independently modulated by a single perceptual dimension. Further analysis of the data from Bizley et al. (2009b) has revealed that indeed, even neurons that are sensitive to multiple perceptual attributes can provide a reliable F0 representation by exhibiting invariant F0 tuning in a particular aspect of its spike response, such as the spike rate within a specific time bin (Walker et al., 2009b). For instance, in the posterior suprasylvian field, information about the location and timbre of sounds was almost exclusively encoded in the early onset phase of the response, whereas sensitivity to stimulus F0 continued later into the sustained response (Fig. 4). This result is consistent with the findings of Ahveninen et al. (2006), who used a combination of fMRI and MEG to investigate the processing of localization and phonetic cues in human auditory cortex. These two cues not only activated different higher order cortical areas, but did so over subtly different time courses. Localization-sensitive cortical voxels were activated 30 ms earlier than those involved in processing phonetic information.

A number of investigations have searched for missing fundamental responses in primary auditory cortex. Since this perceptual feature is dissociable from the spectral content of the sound, such responses would provide strong evidence of a neural representation of the pitch percept. An early investigation aimed to identify neurons that respond to the missing fundamental of harmonic tone complexes in the auditory cortex of awake rhesus monkeys, but this study failed to find such response properties (Fishman et al., 1998), even in monkeys that had been trained to discriminate the pitch of these sounds (Schwarz and Tomlinson, 1990). Bendor and Wang (2005) have performed an extensive search for pitch-selective cortical neurons, in which they presented several types of periodic sounds (including pure tones, tone complexes, and click trains) to awake marmosets. They described a small proportion of neurons in the lateral, low-frequency border of area A1 and R, which exhibit several features of pitch *selectivity*. These neurons could be assigned a characteristic frequency for pure tones, and also responded to missing fundamental tone complexes with F0 at this same frequency. Note that this population of cells differs from the pitch *sensitive* neurons described above, in which pitch tuning to complex sounds did not correlate with characteristic frequency (Bizley et al., 2010). The response of Bendor and Wang's (2005) pitch neurons was related to the temporal regularity (i.e. pitch salience) of sounds, since their spike rates were modulated by the repetition rate of regular click trains, but not by “jittered” click trains. The region in which these neurons were located is homologous to lateral HG in humans, and even though the responses of these apparently pitch-selective neurons were also to some extent sensitive to the intensity and spectral content of sounds (discussed by Tramo et al., 2005), these neurons nevertheless exhibit many of the properties that one would expect to find in a cortical pitch centre. Because pitch is ultimately a perceptual and not a physical property of the sound, conclusive evidence that a particular population of neurons is specialized for pitch processing cannot be based on observations of stimulus response properties alone, but one also needs to demonstrate that the activity of the neurons in question plays a key role in shaping the animal’s subjective perception of the sound. Equally, in human studies cortical activation should be correlated with the perception of pitch rather than the physical properties of the sound.

We have seen that sensitivity to the periodicity of sounds can be found in neurons distributed throughout auditory cortex, and these neurons often also represent other perceptual features. Multiple stimulus dimensions are encoded through spike rates that are tuned to the linear combinations of acoustic features, as well as the independent tuning of spike rates within distinct time bins to a particular stimulus feature. Bendor and Wang (2005) have shown that in the midst of this distributed neural sensitivity to F0, neurons that are more selective for pitch cues and that respond to missing fundamental stimuli can be found clustered in the marmoset homolog of the human lateral HG, in the anterolateral belt. These populations of neurons may constitute a pitch centre in the primate brain, but their precise role in generating pitch percepts should be explored further with experiments that combine behavioural pitch judgments with electrophysiological recording or deactivation.

### 5.5 Pitch in context: higher-order processing of pitch cues

Although an in-depth coverage of melody perception is beyond the scope of the present review, it is worth briefly noting that pitch extraction continues beyond the core and belt of auditory cortex. The activity of higher cortical fields in the parietal and frontal lobes changes selectively in tasks that require more complex pitch judgments, such as those based on the melody (Griffiths et al., 1999; Patterson et al., 2002; Warren and Griffiths, 2003a) or statistics (Gutschalk et al., 2007) of pitches within sound sequences. The analysis of pitch in the context of melody also appears to be lateralized to the right hemisphere in humans (Warrier and Zatorre, 2004; Zatorre et al., 1994). In fact, cortical regions that underlie pitch perception seem to become more widely distributed and lateralized further along the cortical hierarchy (Patterson et al., 2002; Schiavetto et al., 1999; Zatorre et al., 1994).

## 6 Our current understanding and open questions

The body of literature described above suggests that representations of the periodicity of complex sounds are distributed across numerous auditory cortical regions. While some auditory cortical areas seem to play key specialized roles in pitch extraction, these are a part of a wider network that is necessary to explain the range of pitch judgments made by humans and animals. The network of pitch-sensitive regions in auditory cortex may exist to support a variety of periodicity judgments, which are distinguishable based on either function (i.e. pitch directions versus pitch change detection) or stimulus type (i.e. binaural or monaural pitch).

In subcortical structures, cues to periodicity and pitch are often represented by regular temporal patterns of action potentials that are phase-locked to the sound waveform, resulting in periodic trains of spikes. However, the temporal integration windows of neurons widen throughout the ascending auditory pathway, and at the level of A1 the responses of a single neuron are too sluggish to provide phase-locked representations of periodicity within the pitch range. Although temporal representations of pitch may still exist in cortex in the form of temporally precise onset latencies (Fig. 3b), the most commonly observed code for periodicity within cortical neurons is a modulation of spike rates as a function of F0. It is not yet clear how auditory cortical neurons transform the temporal representation of pitch found in the autocorrelation of spikes across subcortical neurons into a monotonic spike rate code. There is evidence, however, that some A1 neurons have multi-peaked frequency tuning that allows them to be sensitive to the harmonic relations of tone complexes. This may indicate a form of spectral template-matching used for pitch extraction at the cortical level. Representations of pitch derived from temporal and spectral processes may later converge onto “pitch neurons” in auditory cortex, or these cues may be processed by separate neural populations, as human lateralization studies suggest.

Functional MRI, electroencephalography and magnetoencephalography studies of the human brain indicate that a cortical region beyond A1, namely lateral Heschl’s gyrus, appears to respond preferentially to periodic sounds, and may be specialized for pitch processing. In the marmoset homologue of this region, a small subgroup of neurons have monotonic spike rate representations of the missing fundamental of tone complexes, supporting a unique role for this region in pitch extraction. But note that these cells also respond vigorously to aperiodic sounds. Therefore, while lateral HG in humans almost certainly plays a key role in pitch perception, questions still remain about how the regional pitch sensitivity observed in fMRI and scalp recording studies may manifest at the single neuron level.

The difficulty in comparing cellular recordings to fMRI or MEG results lies, in part, in the fact that these are almost always carried out in different species. But there is also a more fundamental difference in the type of activity that these techniques measure. Magnetic resonance imaging measures the blood oxygenation level, rather than neural responses directly. A correlation has been demonstrated between this hemodynamic response and local field potentials, suggesting that fMRI results are strongly dependent on dendritic activity (Goense and Logothetis, 2008; Logothetis et al., 2001). Thus, an experimental effect could reflect local processing in the region of interest via synaptic connections. Alternatively, fMRI activity in a region could reflect the activity of synaptic inputs from a projecting neural structure where the process of interest is taking place. Measurements of ionic currents and their resulting magnetic fields are also thought to reflect net dendritic activity. Local field potential measurements can usually be collected during extracellular recording experiments, and the interpretation of these signals in addition to local neural responses may assist the comparison of fMRI results to neural response properties.

There are also major differences in the time scales of the underlying processes measured by these experimental techniques. Blood oxygenation level dependent signals are measured over tens of seconds and thus they reflect the mean rates of membrane potential fluctuations within populations of cells, but are insensitive to fine temporal modulations in spike firing patterns. Extracellular recordings have shown that temporal aspects of neural spike responses, and the local spiking response of single neurons, can carry significant information about the periodicity of sounds that would be unobservable with the very wide temporal or spatial integration of these signals. Thus, fMRI measures only a subset of the neural representations of complex sounds, and it is possible that areas which fail to show pitch modulation in fMRI studies do in fact contain neurons with spatially-delimited or temporally-based spike responses that are highly modulated by the periodicity of sounds. MEG can measure fluctuations in neural activity with millisecond precision, but again these are spatially summed over many millions of active neurons.

Studies of single neuron responses have different limitations. While fMRI and MEG can sample activity across the entire brain, microelectrodes can cover only a limited region of tissue in any one experiment. This is a problem if pitch function is widely distributed, as we propose. Single-unit recordings in animal models also fall short in addressing the role of pitch perception in some higher-level cognitive functions that are arguably unique to humans, such as language and music.

Clearly, each experimental technique has its unique perspective on neural function, and the range of techniques used to investigate pitch processing in humans and animals will be most powerful when they are used in cooperation. This approach should include presenting similar stimuli and asking complementary research questions in studies across species and recording techniques.

In this review, we have also briefly touched upon studies that demonstrate the remarkable plasticity of auditory cortex. The frequency and temporal tuning properties of cortical neurons change dramatically when an animals is engaged in a perceptual task. Presumably, the tuning of cells in higher auditory cortical regions to more complex periodicity cues could also change according to task demands, but this remains to be demonstrated. Additionally, the relative contribution of different regions within a cortical pitch network might also adapt to meet task demands. To understand these processes, studies that record cortical responses while animals actively listen to sounds are essential. The degree of functional divisions across cortical areas may only become apparent in the activity that arises in these pathways when the animal “uses” its cortex to listen carefully to sounds. On the other hand, the cortical responses observed in a highly trained animal may not be generalizable to a population of naive listeners, so a longitudinal aspect to such studies may also prove to be highly informative. Such designs may also offer further insights into the mechanisms of cortical plasticity, which are likely to include a combination of selective excitation (Kilgard and Merzenich, 1998) and inhibition (Otazu et al., 2009).

Beyond Heschl’s gyrus, auditory cortical fields seem to become ever more functionally divergent in their roles in pitch perception. For instance, one fMRI study found that the cortical activation associated with pitch height extends into posterior planum temporale, while a region specifically modulated by pitch chroma changes was found anterior to A1, extending into planum polare (Warren et al., 2003b). The authors interpret these results as evidence for a hierarchical stream of pitch processing that extends beyond primary auditory cortex and is regionally specialized for perceptual functions, including object identification in posterior planum temporale and object-independent, auditory information analysis in more anterior regions. Human studies have also emphasized a division of pitch extraction between the right and left hemispheres. Single unit studies of these types of pitch processes have not yet been carried out in the higher auditory cortex, and the lateralization of pitch extraction in animals is largely unexplored. Addressing these types of questions about the distribution of pitch processing, rather than trying to identify a single “pitch centre” in auditory cortex to account for all pitch judgments, may prove to be a useful redirection of efforts in studies of how pitch is encoded by cortical neurons.
